# Analysis of the Association between CIMP and BRAF^V600E^ in Colorectal Cancer by DNA Methylation Profiling

**DOI:** 10.1371/journal.pone.0008357

**Published:** 2009-12-21

**Authors:** Toshinori Hinoue, Daniel J. Weisenberger, Fei Pan, Mihaela Campan, Myungjin Kim, Joanne Young, Vicki L. Whitehall, Barbara A. Leggett, Peter W. Laird

**Affiliations:** 1 USC Epigenome Center, Keck School of Medicine, University of Southern California, Los Angeles, California, United States of America; 2 Department of Surgery and Department of Biochemistry and Molecular Biology, USC/Norris Comprehensive Cancer Center, Keck School of Medicine, University of Southern California, Los Angeles, California, United States of America; 3 Familial Cancer Laboratory, Queensland Institute of Medical Research, Herston, Queensland, Australia; 4 University of Queensland School of Medicine, Herston, Queensland, Australia; 5 Conjoint Gastroenterology Laboratory, Clinical Research Centre, Royal Brisbane and Women's Hospital Research Foundation, Herston, Queensland, Australia; University of Hong Kong, Hong Kong

## Abstract

A CpG island methylator phenotype (CIMP) is displayed by a distinct subset of colorectal cancers with a high frequency of DNA hypermethylation in a specific group of CpG islands. Recent studies have shown that an activating mutation of *BRAF* (BRAF^V600E^) is tightly associated with CIMP, raising the question of whether BRAF^V600E^ plays a causal role in the development of CIMP or whether CIMP provides a favorable environment for the acquisition of BRAF^V600E^. We employed Illumina GoldenGate DNA methylation technology, which interrogates 1,505 CpG sites in 807 different genes, to further study this association. We first examined whether expression of BRAF^V600E^ causes DNA hypermethylation by stably expressing BRAF^V600E^ in the CIMP-negative, *BRAF* wild-type COLO 320DM colorectal cancer cell line. We determined 100 CIMP-associated CpG sites and examined changes in DNA methylation in eight stably transfected clones over multiple passages. We found that BRAF^V600E^ is not sufficient to induce CIMP in our system. Secondly, considering the alternative possibility, we identified genes whose DNA hypermethylation was closely linked to BRAF^V600E^ and CIMP in 235 primary colorectal tumors. Interestingly, genes that showed the most significant link include those that mediate various signaling pathways implicated in colorectal tumorigenesis, such as *BMP3* and *BMP6* (BMP signaling), *EPHA3*, *KIT*, and *FLT1* (receptor tyrosine kinases) and *SMO* (Hedgehog signaling). Furthermore, we identified CIMP-dependent DNA hypermethylation of *IGFBP7*, which has been shown to mediate BRAF^V600E^-induced cellular senescence and apoptosis. Promoter DNA hypermethylation of *IGFBP7* was associated with silencing of the gene. CIMP-specific inactivation of BRAF^V600E^-induced senescence and apoptosis pathways by *IGFBP7* DNA hypermethylation might create a favorable context for the acquisition of BRAF^V600E^ in CIMP+ colorectal cancer. Our data will be useful for future investigations toward understanding CIMP in colorectal cancer and gaining insights into the role of aberrant DNA hypermethylation in colorectal tumorigenesis.

## Introduction

Aberrant DNA methylation at CpG islands has been widely observed in cancer. Promoter CpG island hypermethylation associated with inactivation of selected tumor suppressor genes appears to be critical in tumors from inception through to maintenance of the tumor phenotype [1]. Distinct subgroups of several types of human cancers have been proposed to have a CpG island methylator phenotype (CIMP) in which an exceptionally high frequency of cancer-specific DNA hypermethylation is found [2], [3]. Although this concept has been controversial [4], we have confirmed the existence of CIMP in colorectal cancer in a large-scale comprehensive study [5].

CIMP in colorectal cancer may arise through a distinct pathway originating in certain subtypes of serrated polyps [6] and is observed in approximately 15% of all colorectal cancer cases [5], [7]. Features associated with CIMP in colorectal cancer include gender (female), proximal location, and poorly differentiated or mucinous histology [3], [5], [7], [8]. Our study using a newly developed CIMP marker panel in colorectal cancers demonstrated that sporadic microsatellite instability (MSI+) occurs as a consequence of CIMP-associated *MLH1* DNA hypermethylation [5]. Furthermore, we found a strong association of CIMP with the presence of an activated mutant form of *BRAF* (BRAF^V600E^) [5]. Both CIMP and *BRAF* mutations have been reported in the earliest stages of colorectal neoplasia: CIMP in apparently normal mucosa of patients predisposed to multiple serrated polyps [9] and *BRAF* mutations in aberrant crypt foci [10].

The RAS-RAF-MEK-ERK signaling pathway is frequently hyperactivated in colorectal cancer. *KRAS* mutations occur most frequently in 30–40% of all colorectal cancers [11] and *BRAF* mutations are present at a frequency of 5–22%, in which the constitutively activated BRAF^V600E^ variant accounts for ∼90% of all the *BRAF* mutations [12]. Mutations in *KRAS* and *BRAF* are generally mutually exclusive, implying equivalent downstream effects in tumorigenesis [13]. However, recent studies have indicated that mutations of these genes might play distinct roles in tumor initiation and/or maintenance [10], [14].

The extremely tight association between BRAF^V600E^ and CIMP raises the question of whether BRAF^V600E^ plays a causal role in the development of CIMP or whether CIMP-associated promoter hypermethylation provides a favorable setting for the acquisition of BRAF^V600E^. In this study, we searched for possible molecular explanations for the association between BRAF^V600E^ and CIMP using the Illumina GoldenGate DNA methylation platform, which examines the DNA methylation status of 1,505 CpG sites located at 807 genes. The GoldenGate DNA methylation assay has been widely used in various studies and is now a standard method for DNA methylation analysis [15]–[24]. Findings obtained from the commercially available “GoldenGate Methylation Cancer Panel I”, in particular, have been validated using various other techniques [15]–[17], [22], [23], making it a reliable source for DNA methylation measurements across 1,505 loci. We were not able to demonstrate a causal contribution of BRAF^V600E^ to CIMP in our cell culture system. However, we identified genes whose DNA hypermethylation was significantly linked with BRAF^V600E^ in primary colorectal tumors. Inactivation of these specific genes in the context of CIMP might drive the acquisition of BRAF^V600E^ in CIMP+ colorectal tumors.

## Results

### Characterization of 21 Human Colorectal Cancer Cell Lines

**Figure 1:**
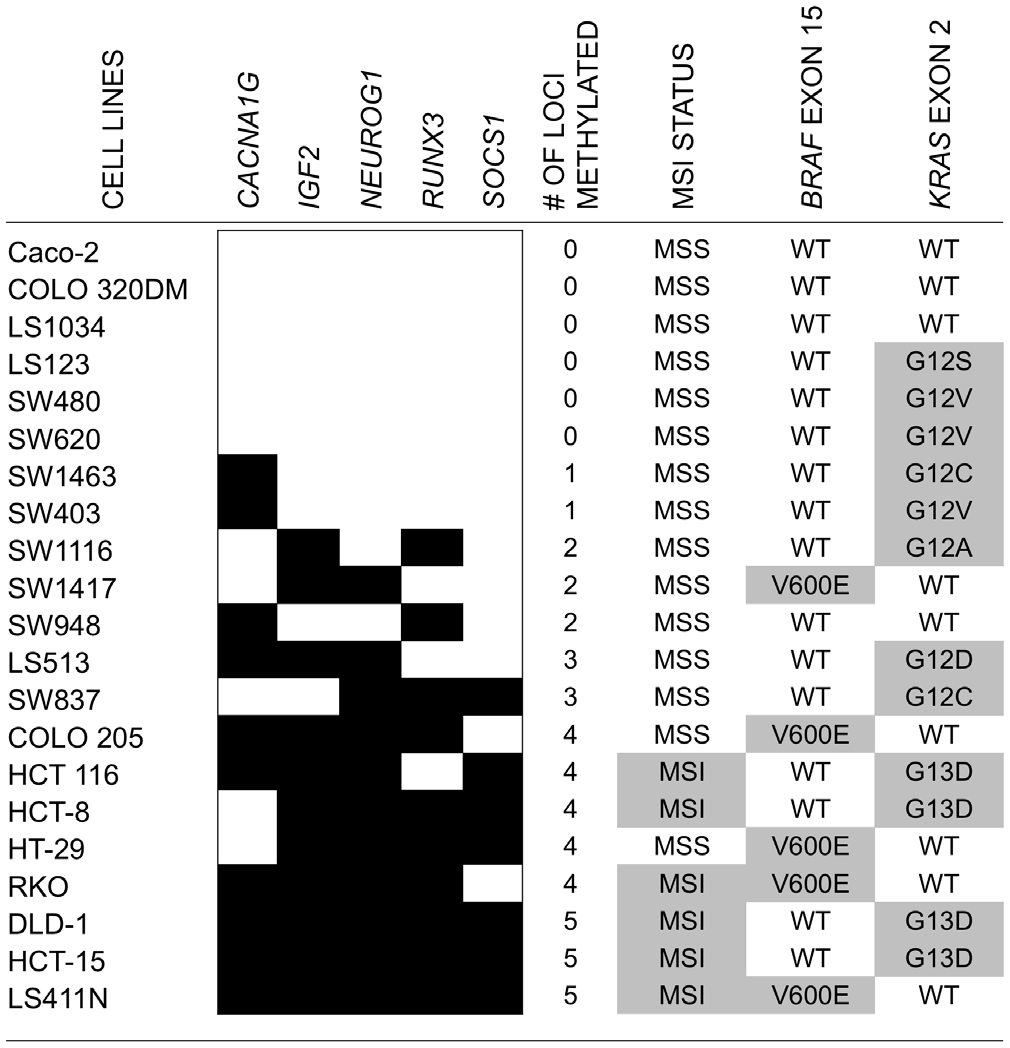
Characteristics of 21 colorectal cancer cell lines. MethyLight was used to assess the DNA methylation status of five CIMP-defining markers. A PMR of ≥10 was used as a threshold for positive methylation. Black boxes indicate PMR ≥10, and white boxes indicate PMR <10. The DNA methylation frequencies of the five CIMP markers increase from top to bottom. Microsatellite instability status for each cell line is listed as microsatellite stable (MSS) or harboring instability (MSI). The mutation status of *BRAF* exon 15 and *KRAS* exon 2 are listed.

We first sought to determine whether expression of BRAF^V600E^ would induce DNA hypermethylation at CpG sites associated with CIMP in an *in vitro* cell culture system. Since primary colonic epithelial cells were not readily available, we screened for colorectal cancer cell lines that do not have substantial DNA methylation at CIMP-defining loci and carry wild-type forms of both *BRAF* and *KRAS*. Such cell lines would serve as suitable systems for the introduction of BRAF^V600E^. We selected 21 colorectal cancer cell lines, characterized their DNA methylation profiles, and determined their *BRAF* and *KRAS* mutation status (Figure 1). We used MethyLight to assess the DNA methylation status of five CIMP-defining markers previously identified in our laboratory [5]. Using a PMR (percent of methylated reference) of ≥10 as a threshold for positive methylation, we identified six cell lines that lacked DNA methylation for all five CIMP-specific markers (Figure 1). To test our hypothesis, we initially chose the *BRAF* and *KRAS* wild-type Caco-2 and COLO 320DM cell lines for their ease in culturing and transfection. However, the study described below is limited to COLO 320DM cells, since we were not able to isolate any stably transfected Caco-2 clones that showed detectable level of BRAF^V600E^ expression (data not shown).

### Stable Transfection of BRAF^V600E^ in COLO 320DM Cells

**Figure 2:**
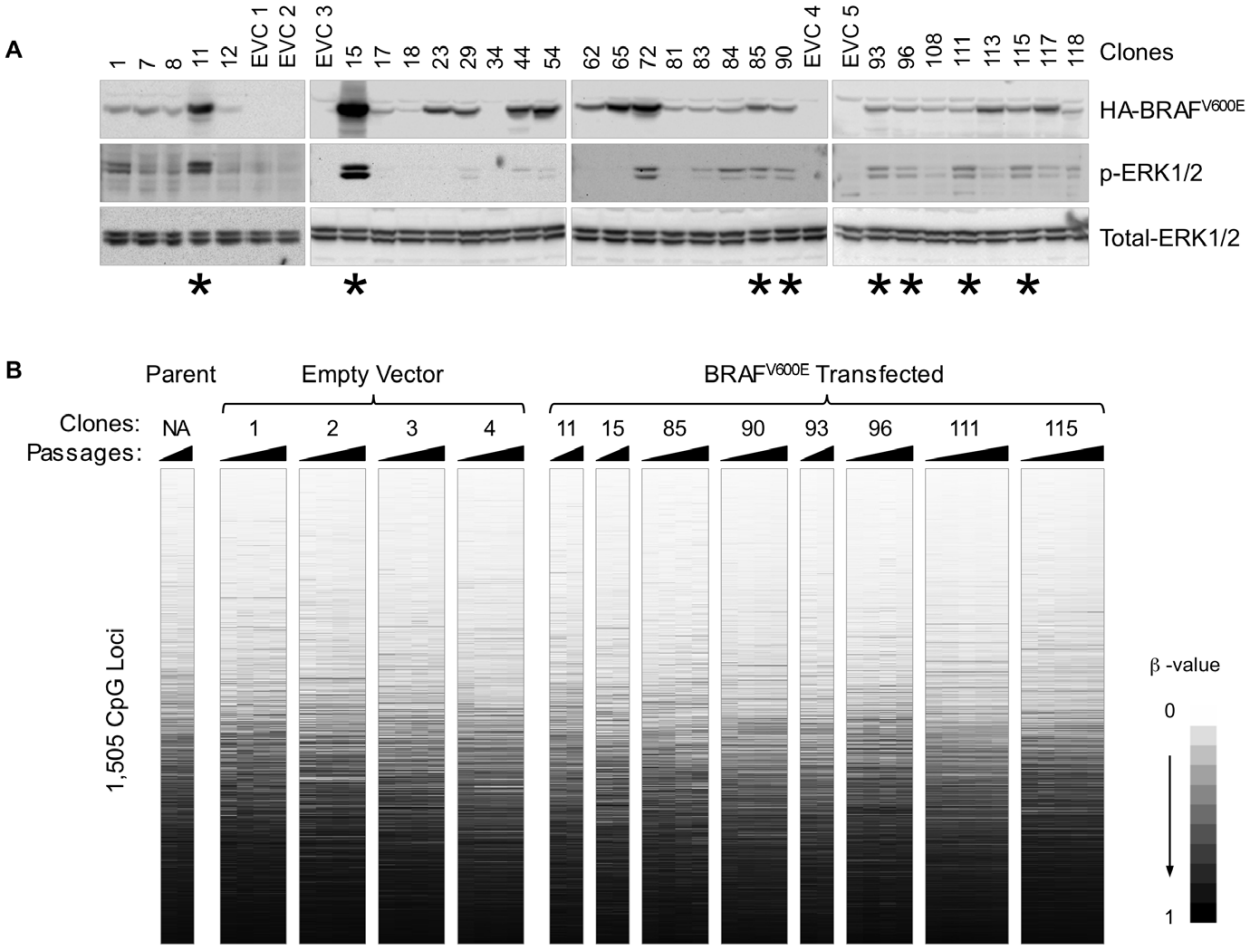
Selection of BRAF^V600E^ stably-transfected clones and their Illumina GoldenGate DNA methylation profiles. (A) Expression of BRAF^V600E^ and ERK1/2 phosphorylation in stably transfected COLO 320DM cells. Blots were probed with the anti-HA antibodies for HA-BRAF^V600E^, anti-phospho-ERK1/2, and anti-ERK1. Asterisks indicate the eight BRAF^V600E^ transfected clones that were subjected to DNA methylation analysis at various cell passages. (B) DNA methylation profiles of untransfected COLO 320DM cells, empty vector and BRAF^V600E^ transfected COLO 320DM clones, as determined by the Illumina GoldenGate DNA methylation assay. The DNA methylation data were scored as β-values as previously defined [16]. Each row corresponds to an individual CpG locus and the data were sorted by average β-value across all samples. Each clone is ordered from left to right in increasing number of passages. EVC: empty-vector transfected clones.

We transfected COLO 320DM cells with an HA-tagged BRAF^V600E^ cDNA and isolated G418-resistant clones. The expression level of BRAF^V600E^ was determined by western blotting using an antibody against the HA epitope (Figure 2A). The activity of BRAF^V600E^ was confirmed by examining the activation of ERK1/2 using an antibody against phosphorylated ERK1/2 (Figure 2A). Eight stably transfected clones exhibiting high expression of BRAF^V600E^, as well as strong activation of ERK1/2, were individually grown in culture, and genomic DNA was isolated at various passages (between 2 and 27) from these clones. Four empty-vector transfected clones (EVCs) were grown in the same conditions and used as controls.

### DNA Methylation Analysis of the BRAF^V600E^ Transfected COLO 320DM Clones

We next determined the DNA methylation status of 1,505 CpG sites located at 807 different genes in each of the eight BRAF^V600E^ clones and four EVCs using the Illumina GoldenGate Methylation Cancer Panel 1 platform (Figure 2B). We found that the DNA methylation β-values across all 1,505 CpG sites in the BRAF^V600E^ transfected clones (regardless of their BRAF^V600E^ expression level) were very similar to those of empty-vector control clones and relatively stable over time. This suggests that there was no overall increase in DNA hypermethylation in BRAF^V600E^ transfected clones in the CpG targets analyzed (Figure 2B).

**Figure 3:**
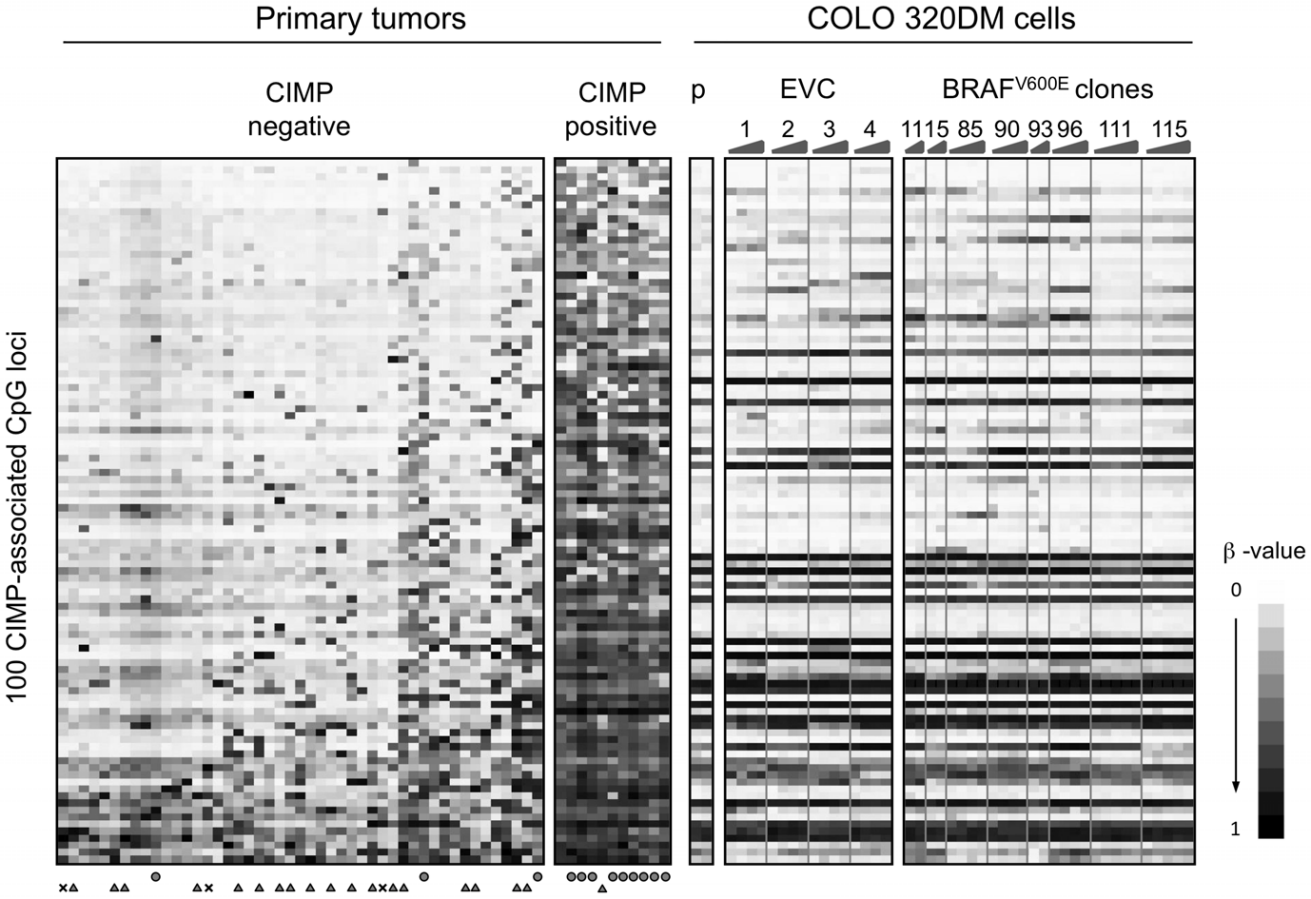
Illumina GoldenGate DNA methylation profiles of CIMP-associated loci. CIMP+ tumors and the CIMP-associated loci in 58 primary tumor samples were defined as described in the Materials and Methods section. Each row corresponds to an individual locus of the 100 locus panel, and the data were sorted by the mean β-value of each locus over all 58 primary tumor samples. Each BRAF^V600E^ transfected clone and EVC is ordered from left to right in increasing number of passages. Tumors with *BRAF* and *KRAS* mutations are indicated by a circle and a triangle, respectively. **X**: mutation status is not available. Each BRAF^V600E^ transfected clone and EVC is ordered from left to right in increasing number of passages. “p” indicates the DNA methylation profiles of parent untransfected COLO 320DM cells.

**Figure 4:**
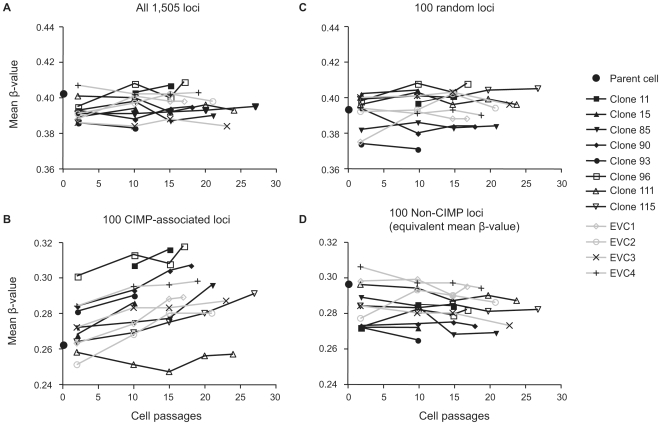
Changes in DNA methylation levels over passages in BRAF^V600E^ and EVC stably-transfected clones. Black lines indicate BRAF^V600E^ expressing clones and gray lines represent empty-vector transfected control clones. Each graphing point represents mean β-values across indicated CpG sites from the Illumina GoldenGate DNA methylation assay at various passages for each clone. (A) All 1,505 CpG loci from the Illumina GoldenGate assay. (B) Only 100 CIMP-associated loci are profiled. (C) One hundred randomly chosen CpG loci. (D) One hundred non-CIMP loci, which show mean β-values similar to the 100 CIMP-associated loci.

We next determined whether the stable expression of BRAF^V600E^ specifically increased the DNA methylation of only CIMP-associated markers in the 1,505 interrogated CpG sites. These sites were determined by screening 58 primary colorectal tumor samples using the Illumina GoldenGate DNA methylation platform (Dataset S1). The mutation status of *BRAF* and *KRAS* in these samples had been determined previously [5] (Table S1). Unsupervised two-dimensional cluster analysis of the DNA methylation β-values revealed a distinct cluster of 11 tumor samples, the majority of which contained BRAF^V600E^ and showed frequent DNA methylation of known CIMP-associated markers, including *CDKN2A, IGF2*, and *MLH1* (data not shown). We defined this subgroup as CIMP-positive tumors (Figure 3). We then identified a total of 100 CpG sites that have significantly higher levels of DNA methylation in CIMP-positive (CIMP+) versus CIMP-negative (CIMP−) tumors (*P*<0.001 after correction for multiple comparisons, see the Materials and Methods section) (Table S2). It should be noted that reactions for three of the MethyLight-based CIMP markers (*CACNA1G*, *NEUROG1*, and *SOCS1*) previously identified in our laboratory are not included in the Illumina GoldenGate Methylation Cancer Panel 1 platform. The *RUNX3* Illumina GoldenGate reactions did not demonstrate CIMP-specific behavior. One possible explanation for this discrepancy could be that these reactions are designed around the transcription start site of *RUNX3* isoform 1, whereas our CIMP-specific *RUNX3* MethyLight reaction is designed at the promoter CpG island of the *RUNX3* isoform 2 [5]. We saw no apparent difference in DNA methylation between BRAF^V600E^ transfected clones and EVCs at these CIMP-associated CpG sites (Figure 3). Interestingly, we observed that the mean DNA methylation β-value of the 100 CIMP-specific loci increased as a function of cell passage (Figure 4A and 4B). However, this increase did not correlate with the levels of BRAF^V600E^ expression and was also observed in cells transfected with the control vector (Figure 4B). This general increase in the mean β-value is specific for CIMP-associated loci, since the mean β-value from several sets of 100 randomly selected CpG sites did not show a similar trend (Figure 4C and 4D). Therefore, we concluded that, although CIMP-associated CpG islands may be prone to acquire DNA methylation in certain culture conditions, BRAF^V600E^ does not specifically induce CIMP in COLO 320DM cells.

### Identification of Genes That Are Significantly Methylated in Colorectal Tumors Harboring BRAF^V600E^

We also considered the alternative hypothesis that promoter methylation of specific gene targets provides a favorable setting for the acquisition of *BRAF* mutation in CIMP+ colorectal cancers. We previously identified the CIMP status and *BRAF* mutation status of 235 primary colorectal tumor samples [5]. We found BRAF^V600E^ in 33 tumors (14.0%); 31 of these were classified as CIMP+ and only 2 as CIMP−. We performed the Illumina GoldenGate DNA methylation assay on these samples, and identified 60 genes, represented by 89 CpG sites, that are significantly methylated (*P*<0.001) in the 33 BRAF^V600E^-positive tumors (Table S3). These genes are candidates for CIMP-specific inactivation, which may closely synergize with the BRAF^V600E^ to promote tumorigenesis.

To validate the data generated using the GoldenGate DNA methylation platform, we analyzed the DNA methylation status of four CIMP-specific genes (*CALCA*, *EPHA3*, *KIT*, and *SLC5A8*) on a subset of four CIMP-positive and 16 CIMP-negative tumors on the Illumina Infinium DNA methylation platform. These four genes were selected because both analytical platforms interrogate the DNA methylation status of the identical CpG dinucleotide. We then examined the concordance of DNA methylation at each of these loci between the two platforms, and found a high correlation coefficient in all cases (*CALCA*: 0.94, *EPHA3*: 0.95, *KIT*: 0.95, *SLC5A8*: 0.86), lending further support to our initial GoldenGate-based DNA methylation screen.

We confirmed the recently observed associations between DNA hypermethylation of *BMP3* and *MCC* with CIMP+ and BRAF^V600E^ in colorectal cancer [25], [26]. We also found CIMP-specific DNA hypermethylation of *BMP6.* The simultaneous epigenetic inactivation of *BMP3* and *BMP6* was shown to be associated with the activation of the RAS-RAF-MEK-ERK signaling pathway in non-small-cell lung cancer [27]. Moreover, we found an association of *SLC5A8* and *TIMP3* DNA methylation with BRAF^V600E^ in our colorectal tumor samples, as had been previously reported in papillary thyroid carcinomas [28]. The functional consequence of DNA hypermethylation of such tumor suppressor genes linked with CIMP+ and BRAF^V600E^ remains speculative [25]–[28].

Furthermore, we found that DNA methylation of *SMO*, a component of Hedgehog (Hh) signaling, was tightly linked to colorectal tumors with BRAF^V600E^ (Table S3). Intriguingly, it has been recently reported that increased expression of *SMO* contributes to colorectal tumorigenesis [29]. However, Arimura et al. also showed that colorectal cancer cell lines harboring BRAF^V600E^, including COLO 205, HT-29 and RKO, did not appear to show expression of *SMO* [29]. Our data indicates that CIMP-specific promoter DNA hypermethylation might be involved in the repression of *SMO* in colorectal tumors carrying BRAF^V600E^ (Table S3).

### Promoter DNA Hypermethylation and Transcriptional Silencing of *IGFBP7* in *BRAF* Mutant CIMP+ Colorectal Cancer

We identified the *IGFBP7* promoter CpG island as a target for DNA methylation in colorectal tumors harboring BRAF^V600E^ (*P* value  = 3.1×10^−9^, Odds ratio  = 12). BRAF^V600E^ has been shown to induce cellular senescence [30]–[32]. Oncogene-induced senescence (OIS) has been recognized as an important tumor suppressor mechanism [33]. The underlying molecular mechanism of BRAF^V600E^-induced senescence and apoptosis has been elucidated in a recent study [34]. It has been demonstrated that expression of *IGFBP7* is both necessary and sufficient to induce senescence and apoptosis mediated by BRAF^V600E^. Intriguingly, *IGFBP7* was shown to be epigenetically silenced by CpG island promoter hypermethylation specifically in primary melanoma samples carrying BRAF^V600E^, indicating that loss of *IGFBP7* expression is critical in the development of BRAF^V600E^-positive melanoma [34].

**Figure 5:**
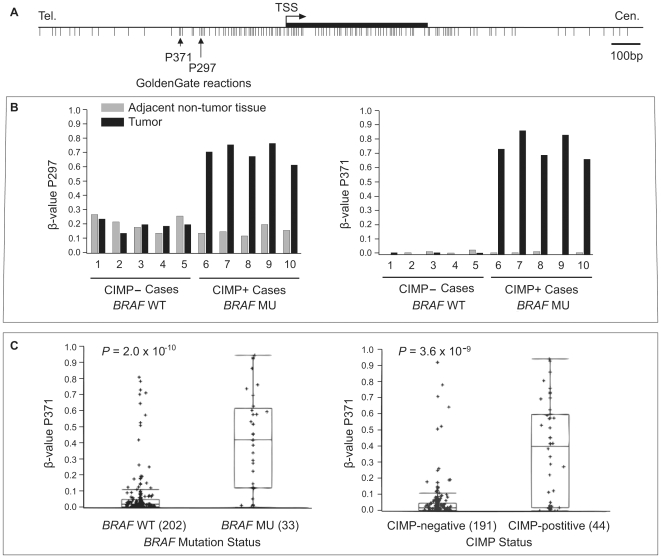
*IGFBP7* promoter DNA methylation in primary colorectal cancers. (A) Genomic map of *IGFBP7* promoter-associated CpG island, transcription start site (TSS) and exon 1 based on the UCSC genome browser (March 2006 assembly). The location of CpG sites interrogated by the Illumina GoldenGate DNA methylation assay is indicated by vertical arrows. (B) DNA methylation levels of the two CpG dinucelotides in the *IGFBP7* promoter CpG island. β-values of each CpG site in 10 tumors (five CIMP− tumors with wild-type *BRAF* and five CIMP+ tumors with mutant *BRAF*, black bars) and adjacent non-tumor tissues (gray bars) are listed. (C) *IGFBP7* promoter DNA methylation box plots of 235 human colorectal tumors stratified by *BRAF* mutation status (left) and CIMP+ status (right) at the *IGFBP7* P371 locus. In the box plots, the ends of the box are the 25th and 75th quartiles. The line within the box identifies the median β-value. The whiskers above and below the box extend to at most 1.5 times the IQR. The CIMP status of each colorectal tumor sample is determined as described in the Materials and Methods section.

**Figure 6:**
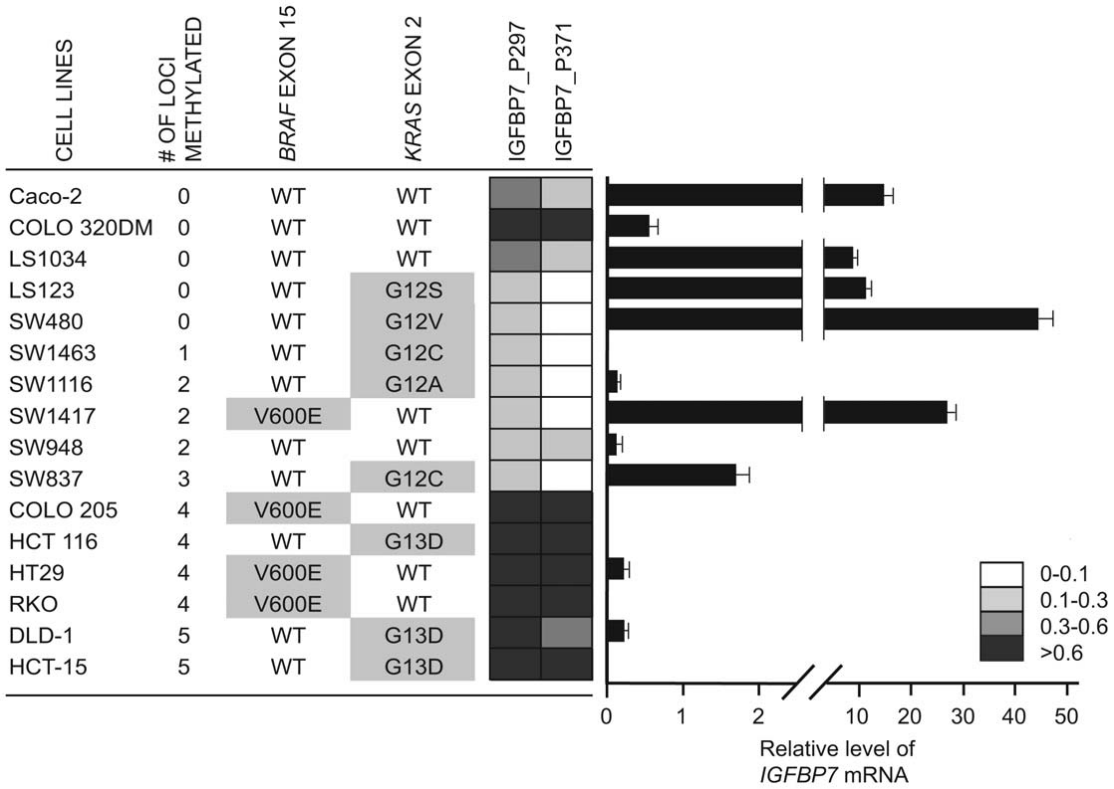
Analysis of DNA methylation and mRNA expression of *IGFBP7* in colorectal cancer cell lines. Quantitative real-time RT-PCR analysis of *IGFBP7* expression. *IGFBP7* expression levels are presented relative to *PCNA* expression. The error bars indicate the standard deviation of technical triplicate measurements. The number of methylated loci among the five CIMP markers and mutation status of *BRAF* and *KRAS* listed in Figure 1 are provided.

The Illumina GoldenGate Methylation Cancer Panel 1 platform contains two *IGFBP7* probes (IGFBP7_P297_F and IGFBP7_P371_F) that interrogate the DNA methylation status of two distinct CpG dinucleotides in the *IGFBP7* promoter CpG island (Figure 5A). We found that these two CpG sites in the *IGFBP7* promoter are cancer-specifically methylated (Figure 5B) and strongly associated with both BRAF^V600E^ (Wilcoxon rank-sum test, *P* value  = 2.0×10^−10^) and CIMP (*P* value  = 3.6×10^−9^) (Figure 5C). It has been reported that colorectal tumors with *KRAS* mutations also show DNA hypermethylation at CIMP-associated markers, albeit at a low frequency, and have high levels of DNA methylation of genes that undergo age-associated DNA hypermethylation. These tumors have been described as CIMP-low or CIMP2 [35], [36]. We did not find an association between *IGFBP7* DNA hypermethylation and *KRAS* mutations when we excluded tumors with mutant *BRAF* (*P* value  = 0.85). In agreement with these observations, we found that DNA hypermethylation of *IGFBP7* is mostly present in colorectal cancer cell lines which harbor BRAF^V600E^ and show frequent DNA methylation of the five-gene CIMP-specific marker panel previously described (Figure 6). Real-time RT-PCR analysis of colorectal cancer cell lines showed that *IGFBP7* mRNA expression was inversely related to DNA hypermethylation, as cell lines with *IGFBP7* hypermethylation showed little or no *IGFBP7* gene expression (Figure 6). Among the CIMP− cells we examined, only COLO 320DM showed DNA hypermethylation of the *IGFBP7* CpG island promoter with minimal level of expression. In retrospect, this unique characteristic of COLO 320DM cells compared to the other CIMP− cell lines might have enabled these cells to tolerate mutant *BRAF* overexpression, and may explain our difficulties in obtaining BRAF^V600E^ expressing clones in other colorectal cancer cell line such as Caco-2.

## Discussion

CIMP in colorectal cancer provides a unique opportunity to study molecular mechanisms that lead to epigenetic changes in cancer and the contributions of these changes in the development of the disease [3], [37]. The distinct features found in CIMP are important clues in understanding this phenotype [3], [5], [7], [8]. Particularly striking is the extremely tight association between CIMP and BRAF^V600E^ [5]. Mechanisms linking epigenetic (CIMP) and genetic (*BRAF* mutation) events and the temporal sequence in which these two events take place have attracted interest [37].

In this study, by using the high-throughput Illumina GoldenGate DNA methylation platform, we investigated the link between CIMP and BRAF^V600E^ in colorectal cancer. We first tested whether expression of BRAF^V600E^ causes DNA hypermethylation by stably expressing BRAF^V600E^ in the CIMP-negative, *BRAF* wild-type COLO 320DM colorectal cancer cell line. We have examined DNA methylation changes in 100 CIMP-associated CpG sites, and found that BRAF^V600E^ is not sufficient to induce DNA hypermethylation at these sites. One caveat of our system is that BRAF^V600E^ could play a role in inducing DNA methylation only early in colorectal tumorigenesis, as *BRAF* mutations have been described at the earliest stage of tumor development [10], [38], [39]. It is possible that a unique set of genetic and/or epigenetic changes that occurred in COLO 320DM cells might have created an unfavorable environment for BRAF^V600E^ to induce DNA hypermethylation. Experiments similar to those described above using Caco-2 cells, which also show CIMP– and carry *BRAF*-wild type, were not successful. We were not able to obtain any stably transfected clones that exhibited detectable levels of BRAF^V600E^ (data not shown). Sustained BRAF^V600E^ expression might be incompatible with Caco-2 cell proliferation due to cellular senescence or apoptosis induced by BRAF^V600E^. It is noteworthy that our RT-PCR analysis showed the robust expression of *IGFBP7*, a mediator of BRAF^V600E^-induced senescence or apoptosis, in Caco-2 cells in contrast to COLO 320DM cells.

Previously, we described CIMP-associated methylation of *MLH1* as the underlying basis for mismatch repair deficiency (MSI+) in sporadic colorectal cancer [5]. Minoo *et al.* reported *MLH1* DNA methylation upon stable transfection of BRAF^V600E^ into the NCM460 cell line [40]. In our system, we did not detect such an increase in *MLH1* DNA methylation (data not shown). Moreover, of the 33 BRAF^V600E^ primary tumors we examined only 42% (14/33) showed *MLH1* DNA hypermethylation. Therefore, BRAF^V600E^ may affect DNA hypermethylation of *MLH1* but only in certain circumstances. Interestingly, in the proposed serrated pathway to CIMP+ tumors, both *BRAF* mutations and CIMP+ have been observed in early precursor lesions, whereas MSI+ has not [6], [10], [41]. Thus, inactivation of *MLH1* might occur at a later stage of tumor development. Minoo and colleagues observed the DNA hypermethylation of *CDKN2A* and 15 other CIMP-associated markers (*IGFBP7* was not examined) in parent NCM460 cells, which limited their ability to study further the role of BRAF^V600E^ inducing CIMP in their experimental system [40].

Intriguingly, we observed that the overall DNA methylation level of the CIMP-specific loci in our stably transfected cells increases as a function of cell passage. It is interesting to note that a selection drug in cultured cells has been described to result in changes in global chromatin structure [42], and a similar process may be associated with our observations here.

In addition, we found relatively large inter-clonal (among different clones) variation in DNA methylation levels in our transfection experiments (Figures 2B and 3), with an average R^2^ correlation calculated based on four EVCs of 0.88±0.01 (± s.d.). Our average intra-clonal (within clones at different passages) R^2^ correlation is 0.97±0.01 and the R^2^ correlation between technical replicates in Illumina GoldenGate DNA methylation analysis is 0.98±0.02 [16]. Consequently, we found some large differences in DNA methylation at several loci even among control clones (Figures 2B and 3). This emphasizes the importance of using multiple clones for this type of studies.

Alternatively, the strong association between CIMP and BRAF^V600E^ might arise if CIMP specifically provides a favorable cellular context for BRAF^V600E^ to promote tumorigenesis. In the second set of experiments, we determined genes whose DNA hypermethylation was tightly linked to BRAF^V600E^ and CIMP+ in colorectal cancer. Intriguingly, we observed CIMP-dependent DNA hypermethylation and transcriptional inactivation of *IGFBP7*, which has been shown to mediate BRAF^V600E^-induced cellular senescence and apoptosis [34]. BRAF^V600E^ has been shown to induce cellular senescence in cultured and primary human cells [30], [31], as well as mouse model [32]. Oncogene-induced senescence (OIS) has been recognized as an important tumor suppressor mechanism [33]. In order for BRAF^V600E^ to promote its oncogenic effects, additional cooperative events are required to bypass senescence [33]. Recently, the molecular basis of BRAF^V600E^-induced senescence and apoptosis has been studied in detail. Wajapeyee et al. identified *IGFBP7* as a mediator of BRAF^V600E^-induced senescence in human primary fibroblasts using a genome-wide shRNA screen. Their subsequent findings suggest that *IGFBP7* expression is both necessary and sufficient to induce senescence and apoptosis in human primary melanocytes and melanoma, respectively. Moreover, they observed loss of *IGFBP7* in primary BRAF^V600E^-positive melanoma samples and concluded that silencing of *IGFBP7* expression is a critical step in the development of a melanoma harboring BRAF^V600E^ [34].

Promoter-associated CpG island DNA hypermethylation of *IGFBP7* has been reported in human colorectal cancer cell lines as well. The DNA methylation inhibitor 5-aza-2′-deoxycytidine has been shown to restore *IGFBP7* expression in colorectal cancer cell lines, indicating that the DNA hypermethylation plays a major role in silencing of this gene in colorectal cancer [43]. However, its association with *BRAF* mutation and CIMP+ status in human colorectal cancers has not been explored. In this study, we found that *IGFBP7* DNA hypermethylation is tumor-specific and tightly associated with colorectal tumors carrying BRAF^V600E^ and exhibiting CIMP. Moreover, we found that *IGFBP7* DNA hypermethylation is associated with loss of expression in CIMP+ colorectal cancer cell lines. CIMP-specific inactivation of BRAF^V600E^-induced senescence and apoptosis pathway by *IGFBP7* DNA hypermethylation might create a favorable context for the acquisition of BRAF^V600E^ in CIMP+ colorectal cancer.

Importantly, *IGFBP7* DNA hypermethylation was not observed in all of the *BRAF* mutant colorectal tumors. Lin *et al*. examined the DNA sequence of the promoter and exonic regions of *IGFBP7* in ten colorectal cancer cell lines. They did not find mutations associated with inactivation of *IGFBP7* in their cell lines [43]. However, an increasing number of genes have recently been reported to be involved in OIS, and cooperation of multiple different signals appears to be critical for OIS [44]. It is therefore possible that CIMP-associated DNA hypermethylation events may impair OIS by affecting other components of the OIS signaling pathway in colorectal cancer.

Additional genes that showed CIMP-specific DNA hypermethylation include those that mediate various signaling pathways implicated in colorectal tumorigenesis (Table S3). The functional consequence of CIMP-specific DNA hypermethylation of such genes will be the subject of future investigations. We found that both *BMP3* and *BMP6* are targeted for CIMP-specific DNA hypermethylation and are strongly linked with BRAF^V600E^. Disruption of the BMP signaling pathway has been proposed to play a role in colorectal tumorigenesis [25]. Concurrent epigenetic inactivation of *BMP3* and *BMP6* was shown to be associated with the hyperactivation of the RAS-RAF-MEK-ERK signaling pathway in non-small-cell lung cancer [27]. Furthermore, receptor tyrosine kinases (RTKs) such as *EPHA3, KIT*, and *FLT1* also showed CIMP-associated DNA hypermethylation (Table S3). Somatic mutations or overexpression of these genes has been implicated in colorectal tumorigenesis, which may involve the activation of the RAS-RAF-MEK-ERK signaling [45]–[50]. The potential inactivation of these genes in CIMP may lead to the development of tumors dependent on oncogenic BRAF-driven hyperactivation of the RAS-RAF-MEK-ERK signaling pathway. Furthermore, we also found that DNA methylation of *SMO* and *HHIP* were closely linked to colorectal tumors with BRAF^V600E^ (Table S3). *SMO* and *HHIP* are involved in the regulation of the Hedgehog (Hh) signaling pathway. It has been demonstrated that elevated expression of *SMO* might contribute to colorectal tumorigenesis through activation of the Wnt signaling pathway in a mouse model and colorectal cancer cell lines [29]. Notably, it appeared that the expression of *SMO* was silenced in colorectal cancer cell lines harboring BRAF^V600E^ [29]. Our data in primary colorectal tumors indicate that the CIMP-specific promoter DNA hypermethylation may result in a different effect of the Hedgehog (Hh) signaling pathway on colorectal tumorigenesis (Table S3).

Our data will be a useful resource for future investigations toward understanding CIMP and the role of aberrant DNA hypermethylation in colorectal tumorigenesis. The inactivation of a senescence pathway by *IGFBP7* DNA hypermethylation in CIMP+ tumors may provide a permissive environment for the acquisition of BRAF^V600E^, thus providing a possible explanation for the link between BRAF^V600E^ and CIMP in colorectal cancer.

## Materials and Methods

### Ethics Statement

This study was conducted according to the principles expressed in the Declaration of Helsinki. The study was approved by the Institutional Review Board of the Royal Brisbane Hospital Human Research Ethics Committee, the Bancroft Centre Ethics Committee and the University of Southern California. All patients provided written informed consent for the collection of samples and subsequent analysis. DNA from these patients was also analyzed in a previous publication [5].

### Cell Culture and Genomic DNA Isolation

Colorectal cancer cell lines were obtained from American Type Culture Collection (Manassas, VA, USA). COLO 320DM cells were grown in DMEM supplemented with 10% FBS, 1 mM glutamine. An empty vector and an HA-tagged BRAF^V600E^ cDNA clone (pMEV-HA, pMEV-BRAF-V599E, Biomyx Technology, San Diego, CA, USA) were transfected into COLO 320DM cells using Lipofectamine 2000 (Invitrogen, Carlsbad, CA, USA). G418 (Sigma-Aldrich, St. Louis, MO, USA) (1 mg/ml) was added 48 hours after transfection and resistant clones were randomly isolated and expanded. Stably expressing clones were maintained in 500 µg/ml of G418. Genomic DNA from each clone was isolated as previously described [51].

### MethyLight Analysis of Five CIMP-Specific Markers in Colorectal Cancer Cell Lines

Genomic DNA was treated with sodium bisulfite and subsequently analyzed by MethyLight as previously described [5], [52]. The primer and probe sequences for the MethyLight reactions were described previously [5]. The results of MethyLight analyses were scored as PMR (Percent of Methylated Reference) values as previously defined [5].

### Mutation Analysis and MSI Status of Colorectal Cancer Cell Lines

Primer sequences and PCR conditions for direct sequencing of *BRAF* at codon 600 in exon 15 and at codons 12 and 13 of *KRAS* in exon 2 were reported previously [13]. The MSI status of each cell line was based on the Sanger Institute Cancer Genome Project (http://www.sanger.ac.uk/) and based on a previous study [53].

### Western Blot Analysis

Whole cell extracts were prepared from each resistant clone at the first passage using CelLytic M Cell Lysis Reagent (Sigma-Aldrich). Equal amounts of protein from whole cell extracts were separated on gradient (4–20%) polyacrylamide gels (Invitrogen) and then transferred to polyvinylidene difluoride (PVDF) membranes (Bio-Rad, Hercules, CA, USA). Blots were probed with the anti-HA antibodies (Roche, Indianapolis, IN, USA) for HA-BRAF^V600E^, anti-phospho-ERK1/2 (Cell Signaling, Beverly, MA, USA), and anti-ERK1 (Santa Cruz Biotechnology, Santa Cruz, CA, USA) followed by incubation with species specific horseradish peroxidase-conjugated secondary antibodies (Santa Cruz). Proteins were visualized using SuperSignal West Pico Chemiluminescent Substrate (Pierce, Rockford, IL, USA).

### Primary Colorectal Tissue Samples

Primary colorectal tissue samples were collected and DNA was extracted as previously described [5]. A 58 sample set included five CIMP+ tumors, five CIMP– tumors and 48 randomly selected tumors, as indicated previously [5]. A 235 sample set included the same 48 randomly selected samples described above along with an additional 187 randomly collected tumors described previously [5]. CIMP status and mutation status of *BRAF* and *KRAS* for each tumor sample was previously determined [5]. *BRAF* mutations and *KRAS* mutations were found in 15% (33/235) and 33% (74/221) of the colorectal tumor samples, respectively. The *KRAS* mutation status of 14 tumor samples was not available. *BRAF* mutations and *KRAS* mutations were mutually exclusive [5].

### DNA Methylation Analysis by the Illumina GoldenGate and Infinium DNA Methylation Platforms

Genomic DNA was bisulfite converted using the EZ-96 DNA Methylation Kit (ZYMO Research, Orange, CA, USA) according to manufacturer's protocol. Illumina GoldenGate DNA methylation analyses were performed as previously described [16] at the USC Epigenome Center Core Facility. Target sequences for the assay and detailed information on each interrogated CpG site and the associated gene on the “GoldenGate Methylation Cancer Panel 1” are described at www.illumina.com. The Illumina Infinium DNA methylation assay was performed following manufacturer's specifications. Detailed information on each interrogated CpG site and the associated gene on the Infinium BeadArray is available at www.illumina.com.

### Identification of CIMP-Associated DNA Methylation Markers Using 58 Primary Colorectal Tumor Samples

For the hierarchical cluster analysis on 58 primary tumor samples shown in Figure 3, we used the β-values obtained from 1,421 reactions (84 X-linked reactions were omitted). We used Euclidian distance and Ward's linkage method to perform the clustering (JMP 6.0 software, SAS Institute, Cary, NC, USA). In order to identify CIMP-associated CpG sites, we performed a *t*-test on the difference in the β-value between the CIMP-positive group (11 tumors) and CIMP-negative group (47 tumors). We selected 100 CpG sites with *P*<0.001 after a correction for multiple-comparison [45] and mean |Δβ|>0.17, the estimated error in β [16].

### Quantitative Real-Time RT-PCR

Total RNA from colorectal cancer cell lines were isolated using RNeasy Mini Kit (QIAGEN GmbH, Hilden, Germany). Reverse transcription reaction was performed using the SuperScript® III First-Strand Synthesis kit (Invitrogen). Quantitative real-time PCR was performed with primers and probe purchased from Applied Biosystems (Assay ID Hs00266026_m1) (Foster City, CA, USA). The raw expression values were normalized to those of *PCNA*.

## Supporting Information

Dataset S1 Raw β-values obtained from the Illumina GoldenGate DNA methylation assay on 58 primary colorectal tumor samples. Samples are labeled with internal IDs.
(1.72 MB XLS)

Table S1 Characteristics of the 58 Primary colorectal tumor samples.
(0.02 MB XLS)

Table S2 One hundred (100) CpG sites that have significantly higher levels of DNA methylation in CIMP-positive versus CIMP-negative tumors. *P* values and difference in mean β-values between CIMP-positive tumors and CIMP-negative tumors are also included.
(0.02 MB XLS)

Table S3 The Illumina GoldenGate DNA methylation loci specifically methylated in colorectal tumors harboring BRAF^V600E^. We performed the Illumina GoldenGate DNA methylation assay on 235 primary colorectal tumor samples, whose CIMP status and *BRAF* mutation status have been determined previously [5]. We dichotomized the DNA methylation β-value (methylated or unmethylated) for each locus. The dichotomization threshold was chosen for each locus using the mean β-value + 3SD (standard deviations) from ten normal mucosal samples. The table lists 89 Illumina GoldenGate DNA methylation targets (of 60 genes) selected with *P*<0.001 (Fisher's exact test) after Bonferroni correction for multiple comparisons. Target CpG sites are sorted based on their *P* values.
(0.03 MB XLS)
